# Proteomic analysis of extracellular vesicles secreted by primary human epithelial endometrial cells reveals key proteins related to embryo implantation

**DOI:** 10.1186/s12958-021-00879-x

**Published:** 2022-01-03

**Authors:** Marina Segura-Benítez, María Cristina Carbajo-García, Ana Corachán, Amparo Faus, Antonio Pellicer, Hortensia Ferrero

**Affiliations:** 1 Fundación IVI, Instituto de Investigación Sanitaria La Fe, Valencia, Spain; 2 Departamento de Pediatría, Obstetricia Y Ginecología, Universidad de Valencia, Valencia, Spain; 3 IVIRMA Rome, Rome, Italy

**Keywords:** Extracellular vesicles, Exosomes, Microvesicles, Embryo implantation, Endometrial cells, Endometrial receptivity, Embryo development, Proteomics, Ultracentrifugation

## Abstract

**Background:**

Successful implantation is dependent on coordination between maternal endometrium and embryo, and the role of EVs in the required cross-talk cell-to-cell has been recently established. In this regard, it has been reported that EVs secreted by the maternal endometrium can be internalized by human trophoblastic cells transferring their contents and enhancing their adhesive and invasive capacity. This is the first study to comprehensively evaluate three EV isolation methods on human endometrial epithelial cells in culture and to describe the proteomic content of EVs secreted by pHEECs from fertile women.

**Methods:**

Ishikawa cells and pHEECs were in vitro cultured and hormonally treated; subsequently, conditioned medium was collected and EVs isolated. Ishikawa cells were used for the comparison of EVs isolation methods ultracentrifugation, ExoQuick-TC and Norgen Cell Culture Media Exosome Purification Kit (*n* = 3 replicates/isolation method). pHEECs were isolated from endometrial biopsies (*n* = 8/replicate; 3 replicates) collected from healthy oocyte donors with confirmed fertility, and protein content of EVs isolated by the most efficient methodology was analysed using liquid chromatography–tandem mass spectrometry. EV concentration and size were analyzed by nanoparticle tracking analysis, EV morphology visualized by transmission electron microscopy and protein marker expression was determined by Western blotting.

**Results:**

Ultracentrifugation was the most efficient methodology for EV isolation from medium of endometrial epithelial cells. EVs secreted by pHEECs and isolated by ultracentrifugation were heterogeneous in size and expressed EV protein markers HSP70, TSG101, CD9, and CD81. Proteomic analysis identified 218 proteins contained in these EVs enriched in biological processes involved in embryo implantation, including cell adhesion, differentiation, communication, migration, extracellular matrix organization, vasculature development, and reproductive processes. From these proteins, 82 were selected based on their functional relevance in implantation success as possible implantation biomarkers.

**Conclusions:**

EV protein cargos are implicated in biological processes related to endometrial receptivity, embryo implantation, and early embryo development, supporting the concept of a communication system between the embryo and the maternal endometrium via EVs. Identified proteins may define new biomarkers of endometrial receptivity and implantation success.

## Background

Establishment of human pregnancy occurs when a competent embryo migrates into the uterus and the blastocyst attaches to the receptive endometrium followed by endometrial invasion, resulting in successful implantation [1]. For that end, changes occur in the endometrium during the mid-secretory phase of menstrual cycle that lead to a receptive phase when blastocyst arrive [2]. However, the probability of pregnancy in one menstrual cycle is only 30% [3] due to different factors, among them the short period of time (4–5 days) in which the endometrium is receptive. This “window of implantation” depends on an adequate transformation regulated by estrogen and progesterone [1, 4]. This endometrial transformation facilitates cell–cell communication, extracellular matrix remodeling, cell adhesion, migration, and invasion [5]. Several studies reported that the endometrium regulates these processes by secreting multiple factors, proteins, and other molecules into the uterine fluid, but the embryo can also modulate the endometrium [2, 6]. Consequently, embryo implantation and pregnancy likely involve a successful crosstalk between the embryo and the maternal endometrium; however, the molecular mechanisms involved in this process are not well understood.

One crucial type of cellular communication occurs via the release of membrane-enclosed compartments commonly known as extracellular vesicles (EVs) [7]. EVs (30–1,000 nm) are secreted by all types of cells into the extracellular environment to transport proteins, DNA, mRNA, miRNA, and other non-coding RNAs and participate in intercellular communication [8–10]. EVs are variable depending on the cell type and the cellular physiological state, and they can modify the phenotype of recipient cells [11, 12]. Numerous reports of EV isolation confirm their presence in a variety of reproductive tissues and biofluids, such as semen [13], follicular fluid [14], oviducts [15], uterine fluid [16], and embryo-conditioned culture medium [17, 18], suggesting a potential role of these EVs in reproduction. Endometrium-derived EVs may be internalized by human trophoblastic cells and transfer their contents to blastocysts, enhancing their adhesive and invasive capacity [19–21], or EVs secreted by embryos may be taken up by the adjacent endometrium for successful implantation [7], suggesting that EVs mediate intercellular communication between embryos and the maternal endometrium. Accordingly, EVs secreted by endometrial cells from patients with recurrent implantation failure decreased blastocyst and hatching rates, total cell number, and invasion capacity in murine embryos, suggesting an important role of these EVs in embryo development [22].

Some studies have described EVs present in uterine fluid, mucus and endometrial epithelial cells containing miRNAs that may contribute to implantation process [16, 23]. Their miRNA content changes during the menstrual cycle, and specific miRNAs involved in implantation processes are enriched in EVs during the receptive phase [24]. Furthermore, proteomic analysis of EVs secreted by a human endometrial adenocarcinoma epithelial cell line (ECC1) suggested that the endometrial epithelium secretes EVs containing proteins involved in cell adhesion, embryo growth, and development, leading to successful implantation and, hence, establishment of pregnancy [5, 20]. Accordingly, a study of the proteomic profile of trophoblast cells treated with EVs secreted by endometrial cells showed that proteins involved in cell adhesion are key factors for embryo implantation [19]. In this regard, detailed characterization of the different molecular cargos of EVs from primary human endometrial epithelial cells (pHEECs) will enable identification of non-invasive markers of successful implantation and, consequently, new therapeutic targets to develop innovative treatments to improve embryo implantation.

Despite the growing interest in the study of EVs and their cargo, to date, the methods to isolate EVs remain suboptimal. EVs from endometrial epithelial cells are secreted into the extracellular environment in small quantities, making isolation of this population of EVs particularly challenging. Ultracentrifugation is the gold-standard method for isolating EVs secreted by endometrial epithelial cells [5, 19, 22, 23]. However, variable quantities of EVs have been recovered using this method, and its results have never been compared with newer and less labor-intensive methods that are based on peptide-mediated affinity or chemical precipitation of EVs from biofluids, such as urine [25], serum [26], or uterine fluid [16].

Therefore, we first evaluated different methods to isolate EVs secreted by endometrial epithelial cells. To elucidate the molecular mechanisms involved in endometrial receptivity and implantation success, we used the most efficient EV isolation method to isolate EVs secreted by pHEECs and analyzed their protein content via proteomic approaches.

## Methods

### Experimental design

To identify the most efficient methodology for isolating EVs secreted by endometrial epithelial cells, EVs were isolated from conditioned culture medium using three methods. The amount of pHEECs obtained from an endometrial biopsy is insufficient to perform the three isolation methods used in this study. Therefore, EVs were isolated from extracellular medium of the endometrial cell line Ishikawa that was hormonally treated for 48 h using (1) a Norgen Cell Culture Media Exosome Purification kit, (2) the commercial reagent ExoQuick-TC, or (3) an ultracentrifugation method. Isolated EVs were characterized by Western blotting (WB), nanoparticle tracking analysis (NTA), or transmission electron microscopy (TEM) (*n* = 3 replicates/isolation method).

**Fig. 1:**
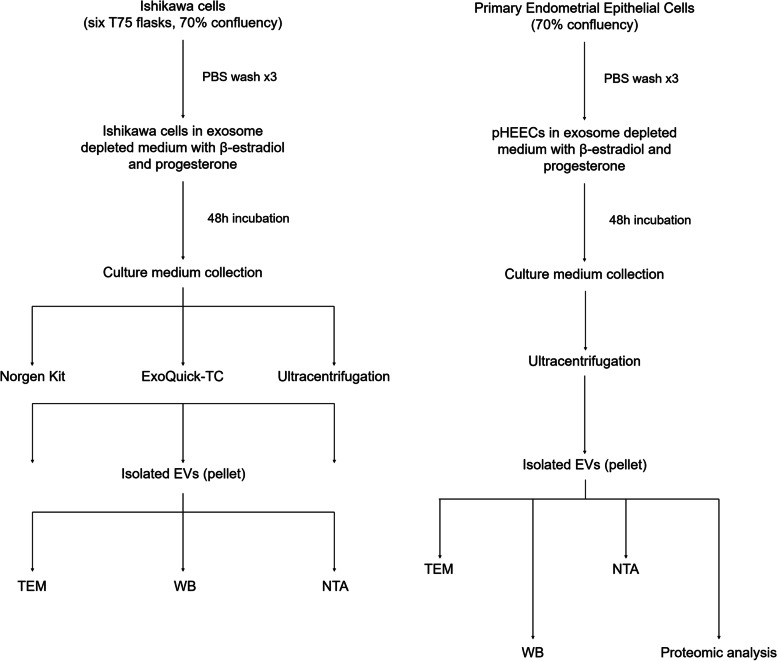
Experimental design. The experimental design included four parts: (1) cell culture of hormone-treated Ishikawa cells and pHEECs; (2) EV isolation by the Norgen kit, ExoQuick-TC reagent, or ultracentrifugation; (3) EV characterization by WB, TEM, or NTA; and 4) proteomic analysis of pHEEC EV cargo. Abbreviations: *WB* Western blot; *TEM* transmission electron microscopy, *NTA* nanoparticle tracking analysis

Once the most efficient isolation method was defined (ultracentrifugation), EVs were isolated from conditioned culture medium of hormonally treated pHEECs and were characterized by WB, NTA, and TEM. Protein content of EVs from pHEECs (n = 8 biopsies/replicate; 3 replicates) was analyzed by liquid chromatography–tandem mass spectrometry (LC–MS/MS) to define proteins and biological functions involved in the implantation process (Fig. 1).

### Sample collection and cell culture

Ishikawa cells (Sigma-Aldrich, MO), a human endometrial adenocarcinoma cell line, were cultured to obtain EVs. These cells have characteristics from both glandular and luminal endometrial epithelium and allow for in vitro culture of large cell numbers that secrete high amounts of EVs [27], in contrast to primary cells whose in vitro culture in large quantities is difficult. Ishikawa cells were cultured in minimum essential medium supplemented with 2 mM glutamine (Thermo Fisher Scientific, USA), 1% non-essential amino acids (Thermo Fisher Scientific, USA), and 5% fetal bovine serum (FBS) (Thermo Fisher Scientific, USA) and incubated at 37 °C with 5% CO_2_ until 70% confluency was reached.

pHEECs were isolated from endometrial biopsies (*n* = 8 biopsies in each of the 3 replicates) collected from healthy oocyte donors (18–35 years old) with confirmed fertility (previous successful pregnancy) and a body mass index of < 30 kg/m^2^ on the day of oocyte retrieval, 36 h after the LH surge. Biopsies were mechanically dissociated using a scalpel and digested with 0.1% collagenase type A1 (Sigma-Aldrich, MO) in Dulbecco’s modified Eagle medium (DMEM) (Thermo Fisher Scientific, USA) at 4 °C overnight. Supernatant containing stromal cells was removed, and the pellet with epithelial cells was incubated with TrypLE Select (Thermo Fisher Scientific, USA) for 1 min to break up the glands. After centrifuging the sample for 5 min at 2,000 rpm, the supernatant was removed, and the pellet was resuspended in culture medium. pHEECs were cultured in DMEM/F12 (Thermo Fisher Scientific, USA) and MCDB 105 (Sigma-Aldrich, MO) (3:1) supplemented with 10% FBS, 5 µg/mL bovine insulin (Sigma-Aldrich, MO), and 0.2% streptomycin and penicillin (Thermo Fisher Scientific, USA) at 37 °C with 5% CO_2_ until 70% of confluency was reached.

### Hormonal treatment

Once confluency was reached, Ishikawa cells and pHEECs were hormonally treated with 10^−8^ M β-estradiol and 10^−7^ M progesterone (Sigma-Aldrich, MO) in culture medium with exosome-depleted FBS (Thermo Fisher Scientific, USA) for 48 h at 37ºC and 5% CO_2_ to mimic the secretory phase of the menstrual cycle. Conditioned culture medium was collected for EV isolation.

### Extracellular vesicle isolation using the Norgen kit

To test the efficiency of the Norgen Cell Culture Media Exosome Purification kit (Norgen Biotek Corp., Canada) for isolating EVs from conditioned culture medium of endometrial epithelial cells, the manufacturer’s protocol was followed. Flowthrough obtained from the mini filter spin column, which contained EVs, was concentrated using 100-kDa molecular weight Amicon Ultra-2 centrifugal filter units (Merck, Germany). Characterization methods, including NTA and TEM, were performed with this concentrated flowthrough; for WB, flowthrough was mixed with RIPA lysis buffer (vol/vol).

### Extracellular vesicle isolation using ExoQuick-TC reagent

The efficiency of the ExoQuick-TC reagent (System Biosciences, USA) to isolate EVs from conditioned culture medium of endometrial epithelial cells was tested by following the manufacturer’s protocol. Conditioned medium was first centrifuged at 3,000 × *g* for 15 min, and the supernatant was again centrifuged at 10,000 × *g* for 30 min. Then, the supernatant was concentrated using 100-kDa molecular weight Amicon Ultra-15 centrifugal filter units (Merck, Germany) and incubated with the ExoQuick-TC reagent overnight at 4 °C. The mixture was centrifuged at 1,500 × *g* for 30 min to pellet EVs. Pellet was resuspended in either PBS (Sigma-Aldrich, MO) for NTA and TEM or in RIPA (Thermo Fisher Scientific, USA) lysis buffer for WB analysis.

### Extracellular vesicle isolation by ultracentrifugation

Ultracentrifugation was assessed for its efficiency in isolating EVs from conditioned culture medium of endometrial epithelial cells. Conditioned culture medium was first centrifuged at 300 × *g* for 10 min at 4 °C, then at 2,000 × *g* for 20 min at 4 °C to remove cells. Supernatant was then transferred to polycarbonate tubes and centrifuged at 10,000 × *g* for 30 min at 4 °C to remove cell debris. Subsequently, supernatant was collected and centrifuged twice at 100,000 × *g* for 70 min at 4 °C to pellet EVs, using a Beckman-Coulter JA-30.50 Ti Rotor. Pellet was resuspended in either PBS or RIPA lysis buffer depending on the characterization method.

### Nanoparticle tracking analysis

NTA of EVs was performed using a NanoSight NS300 instrument (Malvern, Spain) to characterize the size distribution and concentration of EVs isolated from each method. Samples were diluted using PBS at a ratio of 1:100 or 1:1,000 depending on sample concentration, and each experiment was performed in triplicate.

### Protein extraction and Western Blot

To determine the presence of EV protein markers and to validate selected proteins contained in EVs secreted by pHEECs, WB was performed. EV pellets were resuspended in RIPA lysis buffer containing protease inhibitors (Sigma Aldrich, MO) for protein extraction. Protein concentration was then measured by a Bradford protein assay following the manufacturer’s protocol, and samples were separated by SDS-PAGE. WB of samples from each different EV isolation method was conducted to determine the presence of EV markers, including HSP70 (1:200; Santa Cruz Biotechnology), TSG101 (1:500; Abcam), CD9 (1:200; Santa Cruz Biotechnology), and CD81 (1:100; Santa Cruz Biotechnology). The following antibodies were used to determine the expression of selected proteins contained in EVs secreted by pHEECs: ANXA2 (1 µg/mL; Abcam), FN1 (1:2,000; Sigma-Aldrich), ITGAV (1:200; Santa Cruz Biotechnology), PFN1 (1:100; Santa Cruz Biotechnology), and VTN (1:100; Santa Cruz Biotechnology).

Antibodies were detected using a chemiluminescence detection system (Thermo Fisher Scientific, USA), and bands were visualized on an Amersham Imager 680 system. Quantification of protein levels was performed using ImageJ software, and all protein expression was normalized to β-actin (1:2,000; Santa Cruz Biotechnology). HeLa cell lysate (Santa Cruz Biotechnology) was used as a positive control for the expression of HSP70, CD9, and TSG101, and Jurkat cell lysate (Santa Cruz Biotechnology) was used as a positive control for the expression of CD81.

### Transmission electron microscopy

To analyze EV morphology, EVs were resuspended in PBS, and 6 μL were placed on a carbon-coated grid and contrasted with 2% uranyl acetate. EVs were then observed under an FEI Tecnai G2 Spirit BioTwin (Thermo Fisher Scientific, Oregon, USA) transmission electron microscope. Images obtained from each sample were examined by 3 observers, and artifacts were quantified based on their colour, size and shape.

### Proteomic analysis

To characterize the protein cargo of pHEEC-derived EVs and associated biological functions involved in the implantation process, samples were quantified, and 15 µg of protein were digested by standard methods [28]. Subsequently, 4 µg of digested peptides were analyzed for 120 min with an Ekspert nanoLC 425 (Eksigent) coupled to a 6600plus TripleTOF, SCIEX, as previously described [29].

### Protein identification and analysis

ProteinPilot default parameters were used to generate a peak list directly from 6600 plus TripleTOF wiff files. The Paragon algorithm of ProteinPilot v 5.0 was used to search the Swissprot_200601.fasta (562,246 proteins) database with the following parameters: trypsin specificity, cys-alkylation, taxonomy restricted to *Homo sapiens*, and the search effort set to rapid with false discovery rate (FDR) analysis.

For proteomic data analysis, identified duplicates and contaminants were excluded from the list of each sample, and only proteins detected in at least two of the replicates were considered. The Gene Ontology (GO) database was considered through the PANTHER tool, and enrichment networks were identified using geneMANIA and Cytoscape tools.

### Statistical analysis

GraphPad Prism 6.0 was used for statistical analyses and graphics generation. Data are presented as mean ± standard deviation (SD). For WB experiments, NTA, and Bradford assay results, a one-way analysis of variance test was performed to analyze protein expression, protein concentration, size distribution, and concentration of nanoparticles. A *P* value of < 0.05 was considered to be statistically significant.

## Results

### Size distribution and concentration of EVs isolated with different methods

**Fig. 2:**
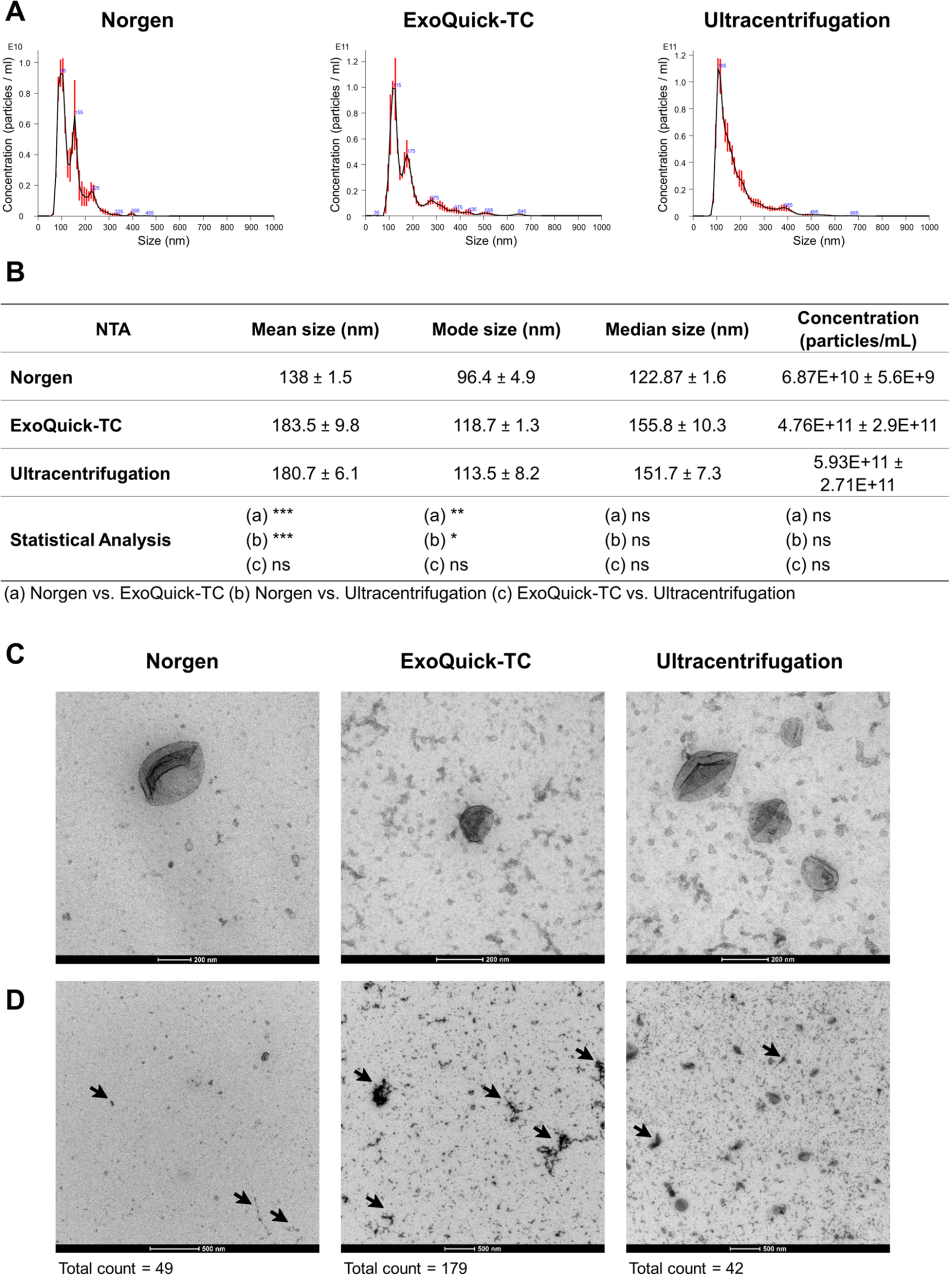
Size distribution, concentration, and morphology of EVs secreted by Ishikawa cells isolated by different methodologies. **A** Representative set of NTA plots of EVs isolated by the Norgen kit, the ExoQuick-TC reagent, or ultracentrifugation, with a dilution factor of 1:100 for Norgen-isolated samples and 1:1,000 for samples isolated by ExoQuick-TC and ultracentrifugation. **B** Summary table of the size distribution and concentration data of isolated nanoparticles. **C** Representative set of TEM images showing the morphology of EVs isolated by the three methodologies with a 200-nm and **D** 500-nm magnification, with total counts of artifacts present in the samples, arrowheads show artifacts; *n* = 3 replicates/method; ***P < 0.001; **P < 0.01; *P < 0.05; ns, not significant. Data are presented as Mean ± SD

To confirm that all three methodologies isolate EVs from the culture medium of endometrial epithelial cells, an NTA, which shows the size distribution and concentration of nanoparticles, was performed in EVs isolated from the extracellular medium of Ishikawa cells. Although the size distribution of nanoparticles isolated by all three methodologies was similar (50–200 nm), greater heterogeneity was observed in nanoparticles isolated by the Norgen and ExoQuick-TC methods (Fig. 2A). Specifically, mean and mode sizes of nanoparticles isolated by the Norgen method were statistically smaller (mean, 138 ± 1.5 nm; mode, 96.4 ± 4.9 nm) than particles isolated by ExoQuick-TC (mean, 183.5 ± 9.8 nm; mode, 118.7 ± 1.3 nm) and ultracentrifugation (mean, 180.7 ± 6.1 nm; mode, 113.5 ± 8.2 nm). Nanoparticles isolated by ExoQuick-TC and ultracentrifugation methods showed similarities in mean, mode, and median sizes (Fig. 2B). In addition, our results showed that the concentration of nanoparticles was lower in Norgen samples (6.8E + 10 ± 5.6E + 9 particles/mL) than in ExoQuick-TC (4.7E + 11 ± 2.9E + 11 particles/mL) and ultracentrifugation (5.9E + 11 ± 2.7E + 11 particles/mL) samples, although no statistically significant differences were observed (Fig. 2B).

### Morphology of EVs isolated with different methods

To visualize Ishikawa cell EVs and evaluate their morphology, TEM was used. TEM showed that EVs from the three different isolation methods presented typical cup-shaped vesicle structures, which were heterogeneous in diameter and ranged between 50 and 200 nm (Fig. 2C). However, a higher concentration of EVs and fewer amounts of artifacts were observed in samples isolated by ultracentrifugation compared to samples isolated by Norgen and ExoQuick-TC, respectively (Fig. 2D).

### Protein marker evaluation of EVs isolated with different methods

**Fig. 3:**
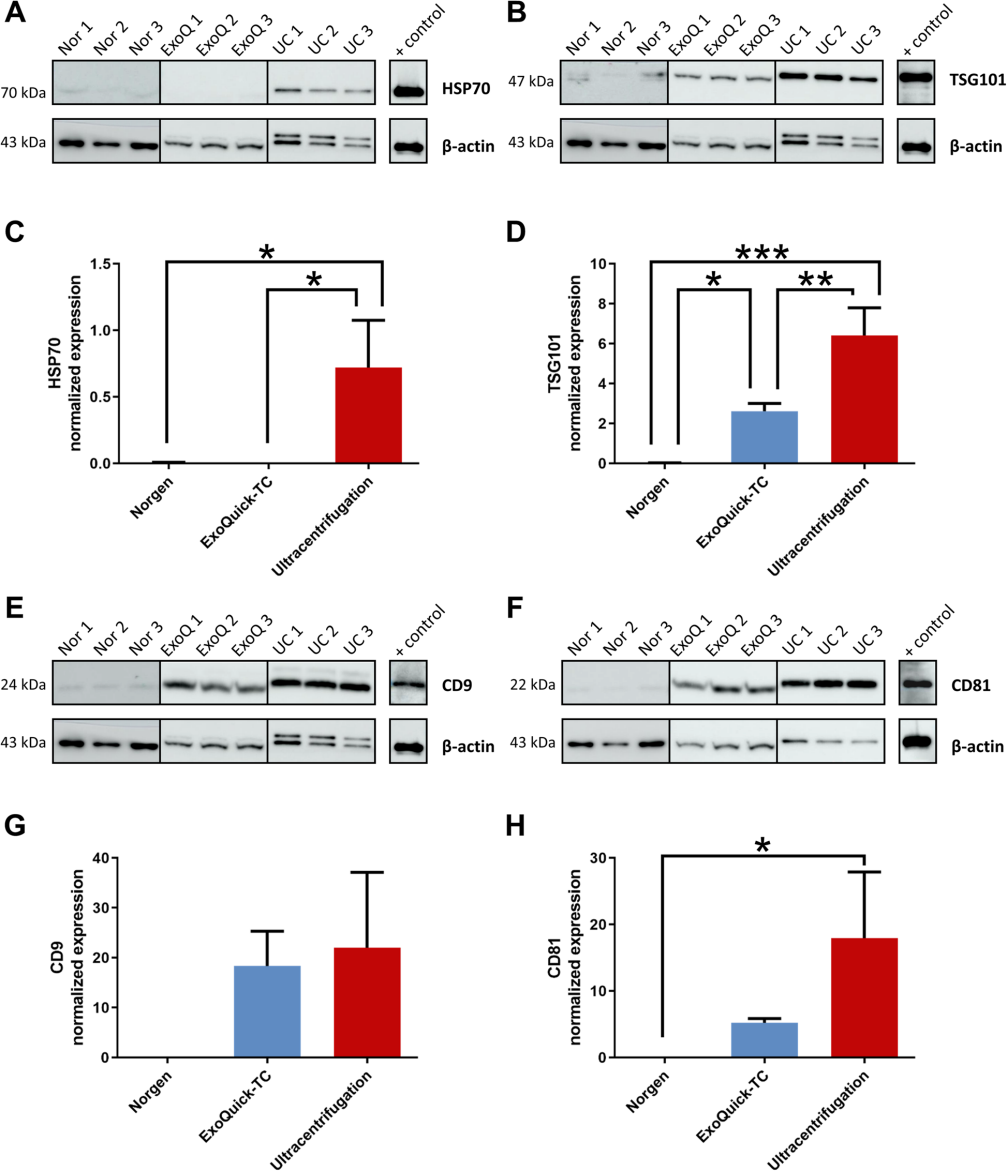
Evaluation of EV protein marker expression. Representative images of Western blots for **A** HSP70 (70 kDa), **B** TSG101 (47 kDa), **E** CD9 (24 kDa), and **F** CD81 (22 kDa) and quantitative protein expression of **C** HSP70, **D** TSG101, **G** CD9, and **H** CD81 in EVs secreted by Ishikawa cells and isolated by the Norgen kit, ExoQuick-TC reagent, or ultracentrifugation (*n* = 3 replicates/method); ***P < 0.001, **P < 0.01, *P < 0.05

To evaluate the presence of Ishikawa cell EVs in samples isolated by the three different methodologies, the expression of EV cytosolic protein markers (TSG101 and HSP70) and membrane protein markers (CD9 and CD81) were examined in EVs isolated from Ishikawa cells (Fig. 3A, B, E and F). A Bradford assay was performed to quantify total protein in each sample. Protein concentration was significantly higher in samples isolated by the Norgen (106 ± 20.6 µg) and ExoQuick-TC (195.9 ± 94.6 µg) methods than in samples isolated by ultracentrifugation (20.7 ± 4.5 µg) (*P* = 0.0022 and *P* = 0.0328, respectively). However, EVs isolated by the Norgen and ExoQuick-TC methods demonstrated low expression for all four EV markers (Fig. 3). Our results showed that expression of the HSP70 marker was statistically higher in EVs isolated by ultracentrifugation than in EVs isolated by the Norgen and ExoQuick-TC methods (*P* = 0.0126 and *P* = 0.0122, respectively), which showed weak or no expression (Fig. 3A and C). Protein expression of the EV marker TSG101 in EVs isolated by ultracentrifugation was statistically higher than in EVs isolated by the Norgen and ExoQuick-TC methods (*P* = 0.0002 and *P* = 0.0033, respectively); TSG101 was also observed to be statistically higher in EVs isolated by the ExoQuick-TC method than in EVs isolated by the Norgen method (*P* = 0.0203) (Fig. 3B and D). CD9 expression was lower in EVs isolated by the Norgen method than in EVs isolated by ExoQuick-TC and ultracentrifugation methods, although no statistically significant differences were observed (*P* = 0.1258 and *P* = 0.0693, respectively) (Fig. 3E and G). Accordingly, CD81 expression was higher in EVs isolated by ultracentrifugation than in EVs isolated by the other two methodologies, although statistically significant differences were only observed when comparing with the Norgen method (*P* = 0.0207) (Fig. 3F and H).

### Characterization of EVs secreted by pHEECs

**Fig. 4:**
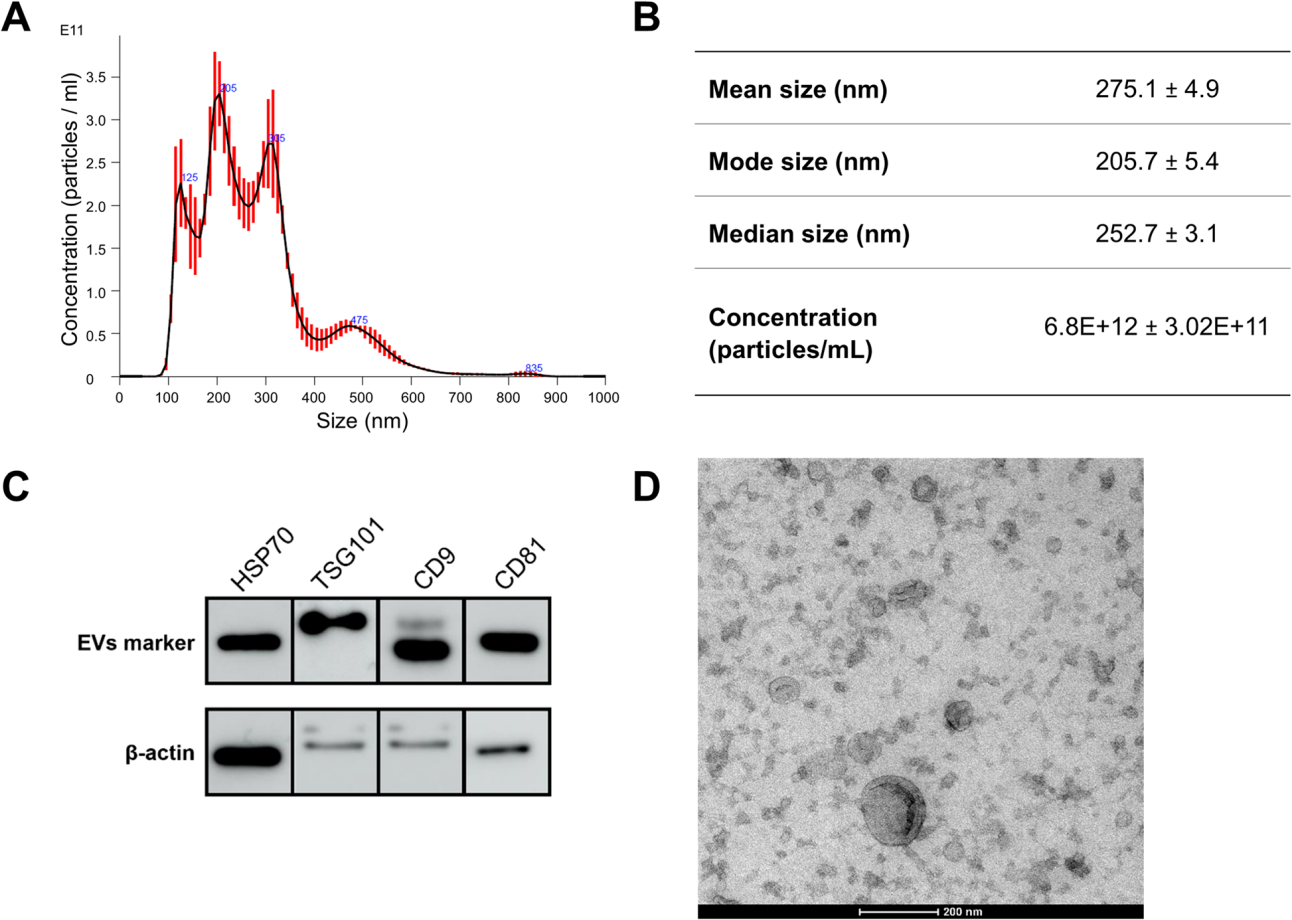
NTA, WB, and TEM characterization of EVs secreted by pHEECs. **A** Representative image of an NTA plot displaying the size distribution of nanoparticles and **B** summary table of the size distribution and concentration data of EVs isolated from pHEECs. **C** Representative images of a Western blot for the EV markers HSP70 (70 kDa), TSG101 (47 kDa), CD9 (24 kDa), and CD81 (22 kDa). **D** Representative image showing the morphology of EVs, with a magnification of 200 nm. Data are presented as Mean ± SD

Ultracentrifugation performed the best to efficiently isolate EVs from cultured endometrial epithelial cells. As such, EVs secreted by pHEECs were isolated by ultracentrifugation. These EVs were characterized by NTA, WB of EV protein markers, and TEM (Fig. 4). Size distribution revealed a heterogeneous population of nanoparticles, with a mean size of 275.1 ± 4.9 nm and a concentration of 6.8E + 12 ± 3.02E + 11 particles/mL (Fig. 4A and B). In addition, protein quantification of EV markers positively identified HSP70, TSG101, CD9, and CD81 expression in EVs isolated from pHEECs (Fig. 4C). Visualization of EVs showed typical cup-shaped vesicle structures with diameters between 50 and 300 nm, corroborating typical EV structural characteristics (Fig. 4D).

### Protein cargo of EVs secreted by pHEECs

**Fig. 5:**
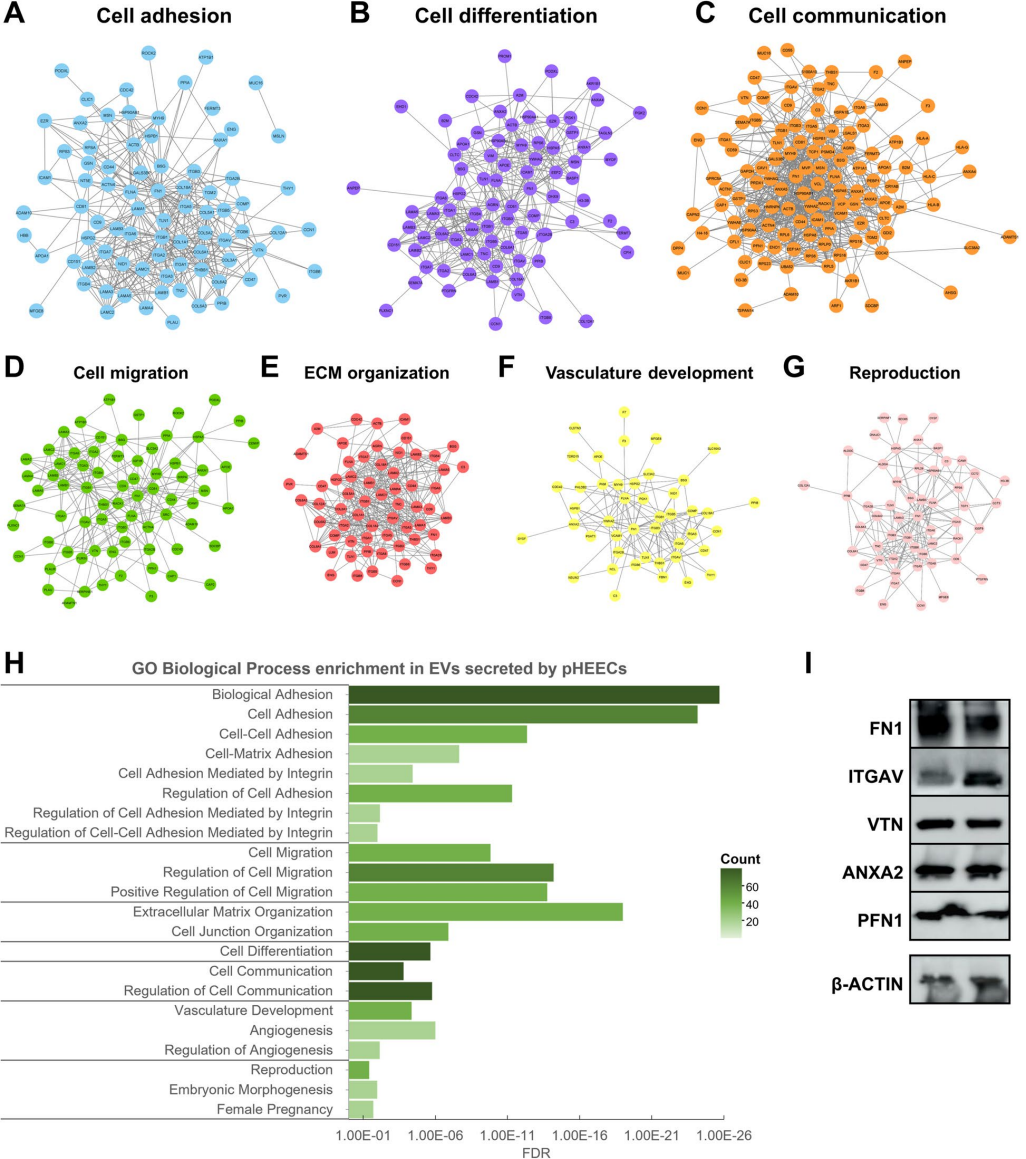
Proteomic analysis of EV cargo secreted by pHEECs. Physical interaction networks for proteins involved in GO biological processes, which could be clustered into **A** cell adhesion, **B** cell differentiation, **C** cell communication, **D** cell migration, **E** ECM organization, **F** vasculature development, and **G** reproduction processes. **H** Enrichment analysis of GO biological processes in EV protein cargo; intensity of the color represents the number of proteins from our data set associated with each process. **I** Representative image of Western blot for validation of the presence of proteins FN1, ITGAV, VTN, ANXA2, and PFN1. FDR, adjusted *P* value; count, number of proteins enriched in each biological process

To define protein content in EVs secreted by pHEECs and associated biological processes involved in the implantation process, an analysis of protein cargo was performed using LC–MS/MS proteomic analysis. Results showed the presence of 218 proteins contained in isolated EVs. Functional enrichment analysis of these proteins revealed several biological processes involved in embryo implantation, which could be clustered in cell adhesion (Fig. 5A), cell differentiation (Fig. 5B), cell communication (Fig. 5C), cell migration (Fig. 5D), extracellular matrix (ECM) organization (Fig. 5E), vasculature development (Fig. 5F), and reproductive processes (Fig. 5G). Biological processes included in each clustering group and protein count are described in Fig. 5H. After a literature review of these proteins, 82 were selected for their functional relevance in implantation success and were divided into three groups depending on their involvement in implantation (Table 1).

**Table 1:** Protein cargo of EVs secreted by pHEECs related to implantation success

UniProt accession	Gene name	Protein description	References (PMID)
**Endometrial receptivity**			
P01023	*A2M*	Alpha-2-macroglobulin	7,533,769, 1,380,439
P60709	*ACTB*	Actin, cytoplasmic 1	29,093,507, 23,638,092
Q9UHI8	*ADAMTS1*	A disintegrin and metalloproteinase with thrombospondin motifs 1	23,751,571, 16,941,747
O00468	*AGRN*	Agrin	18,561,091, 16,885,540
P07355	*ANXA2*	Annexin A2	31,738,896, 33,010,173
P09525	*ANXA4*	Annexin A4	22,999,554
P08758	*ANXA5*	Annexin A5	18,793,766
P61769	*B2M*	Beta-2-microglobulin	23,555,582, 16,251,498
Q08722	*CD47*	Leukocyte surface antigen CD47	21,429,575, 26,839,151
P08174	*CD55*	Complement decay-accelerating factor	23,427,180, 29,153,978
P60953	*CDC42*	Cell division control protein 42 homolog	31,606,658, 20,357,266
O15551	*CLDN3*	Claudin-3	26,340,953, 23,909,989
P12109	*COL6A1*	Collagen alpha-1 (VI) chain	7,780,011
P12110	*COL6A2*	Collagen alpha-2 (VI) chain	7,780,011
P12111	*COL6A3*	Collagen alpha-3 (VI) chain	7,780,011
P49747	*COMP*	Cartilage oligomeric matrix protein	23,555,582
P02511	*CRYAB*	Alpha-crystallin B chain	9,237,261, 20,631,402
P27487	*DPP4*	Dipeptidyl peptidase 4	28,523,980, 29,264,977
P17813	*ENG*	Endoglin	26,802,878
P13726	*F3*	Tissue factor	23,555,582
P21333	*FLNA*	Filamin-A	25,557,137
P05362	*ICAM1*	Intercellular adhesion molecule 1	21,448,983
P56199	*ITGA1*	Integrin alpha-1	32,676,925, 9,130,881
P17301	*ITGA2*	Integrin alpha-2	8,288,018, 9,130,881
P23229	*ITGA6*	Integrin alpha-6	8,288,018, 9,130,881
P05556	*ITGB1*	Integrin beta-1	32,676,925, 9,130,881
P11047	*LAMC1*	Laminin subunit gamma-1	32,980,684, 32,676,925
Q08380	*LGALS3BP*	Galectin-3-binding protein	30,021,913
P26038	*MSN*	Moesin	14,613,898
P15941	*MUC1*	Mucin-1	29,929,546, 25,747,132
Q8WXI7	*MUC16*	Mucin-16	23,555,582
Q99102	*MUC4*	Mucin-4	33,193,109
O43490	*PROM1*	Prominin-1	27,166,505
P21980	*TGM2*	Protein-glutamine gamma-glutamyltransferase 2	18,561,091
Q9Y490	*TLN1*	Talin-1	33,711,384
**Embryo implantation**			
P15121	*AKR1B1*	Aldo–keto reductase family 1 member B1	17,640,989
P04083	*ANXA1*	Annexin A1	32,403,233, 31,940,782
P02647	*APOA1*	Apolipoprotein A-I	27,567,428, 21,676,393
P35613	*BSG*	Basigin	9,501,026, 12,214,805
P01024	*C3*	Complement C3	28,283,674
O00622	*CCN1*	CCN family member 1	17,171,641
P16070	*CD44*	CD44 antigen	29,846,687
P13987	*CD59*	CD59 glycoprotein	7,576,125, 32,973,817
P06733	*ENO1*	Alpha-enolase	24,628,430
Q14512	*FGFBP1*	Fibroblast growth factor-binding protein 1	26,764,347
P02751	*FN1*	Fibronectin	16,621,928, 12,476,048
P98160	*HSPG2*	Basement membrane-specific heparan sulfate proteoglycan core protein	17,442,708
P26006	*ITGA3*	Integrin alpha-3	29,846,687
P06756	*ITGAV*	Integrin alpha-V	10,775,178, 30,929,718
P05106	*ITGB3*	Integrin beta-3	10,775,178, 30,929,718
Q16787	*LAMA3*	Laminin subunit alpha-3	33,715,134, 29,846,687
Q13751	*LAMB3*	Laminin subunit beta-3	33,715,134
Q13753	*LAMC2*	Laminin subunit gamma-2	33,715,134
Q08431	*MFGE8*	Lactadherin	24,424,369
P14543	*NID1*	Nidogen-1	16,607,619
P07737	*PFN1*	Profilin-1	32,466,630
P00749	*PLAU*	Urokinase-type plasminogen activator	21,075,828
P62937	*PPIA*	Peptidyl-prolyl cis–trans isomerase A	32,580,158
Q06830	*PRDX1*	Peroxiredoxin-1	33,860,638
P20742	*PZP*	Pregnancy zone protein	14,580,373
P37802	*TAGLN2*	Transgelin-2	30,702,937
P07996	*THBS1*	Thrombospondin-1	29,846,687
P24821	*TNC*	Tenascin	29,846,687
P08670	*VIM*	Vimentin	30,735,538
P04004	*VTN*	Vitronectin	18,026,832
P62258	*YWHAE*	14–3-3 protein epsilon	33,634,576
P63104	*YWHAZ*	14–3-3 protein zeta/delta	17,537,306
**Early embryo development**			
O14672	*ADAM10*	Disintegrin and metalloproteinase domain-containing protein 10	31,373,105, 12,354,787
P15144	*ANPEP*	Aminopeptidase N	28,859,152
P05023	*ATP1A1*	Sodium/potassium-transporting ATPase subunit alpha-1	21,791,182, 31,405,390
P05026	*ATP1B1*	Sodium/potassium-transporting ATPase subunit beta-1	17,317,668
P48509	*CD151*	CD151 antigen	33,450,381
Q00610	*CLTC*	Clathrin heavy chain 1	33,718,352
P14384	*CPM*	Carboxypeptidase M	14,614,042
P22352	*GPX3*	Glutathione peroxidase 3	33,322,741, 24,279,306
P07900	*HSP90AA1*	Heat shock protein HSP 90-alpha	33,856,486, 9,118,006
P08238	*HSP90AB1*	Heat shock protein HSP 90-beta	33,856,486, 9,118,006
O75367	*MACROH2A1*	Core histone macro-H2A.1	19,734,898
Q13421	*MSLN*	Mesothelin	33,216,405
P63244	*RACK1*	Receptor of activated protein C kinase 1	25,143,135
P08865	*RPSA*	40S ribosomal protein SA	25,800,042
P62987	*UBA52*	Ubiquitin-60S ribosomal protein L40	30,135,083

Regarding endometrial receptivity, we detected proteins involved in cell adhesion, differentiation, and communication processes, such as annexins (ANXA2, ANXA4, and ANXA5), ACTB, and FLNA. Proteins found in EVs included in this group also participate in cell adhesion and communication, including integrins (ITGA1, ITGA2, ITGA6, and ITGB1), mucins (MUC1, MUC4, and MUC16), or other adhesion proteins (AGRN, CD55, CD47, CLDN3, DPP4, LGALS3BP, MSN, TGM2, and TLN1) as well as collagen VI chains ⍺1, ⍺2, and ⍺3, which also participate in ECM organization. Proteins involved in ECM, such as A2M, ADAMTS1, or COMP, were also identified in EVs from pHEECs. In addition, we detected proteins participating in cell differentiation, including B2M, CDC42, ENG, ICAM1, PROM1, or LAMC1. This group also included other proteins (e.g., F3 or CRYAB) (Table 1).

Proteins involved in the embryo implantation process itself were related to GO reproductive processes, such as ANXA1, CCN1, PZP, or VTN. There were also adhesion proteins, such as integrins ITGA3, ITGAV, and ITGB3, and laminin 5 subunits LAMA3, LAMB3, and LAMC2. Other detected cell adhesion-related proteins included APOA1, CD44, FN1, NID1, PLAU, PPIA, or TNC. We also detected cell differentiation-related proteins (AKR1B1, BSG, C3, HSPG2, TAGLN2, or VIM), communication-related proteins (CD59, ENO1, FGFBP1, PFN1, and PRDX1), and angiogenesis-related proteins (MFGE8, THBS1, YWHAE, or YWHAZ) (Table 1).

Finally, proteins involved in early embryo development included ANPEP, CLTC, CPM, GPX3, MACROH2A1, HSP90AA1, or HSP90AB1, which are related to cell differentiation, and other proteins, such as ADAM10, ATP1A1, ATP1B1, CD151, MSLN, RACK1, RPSA, or UBA52, which are related to cell adhesion and communication (Table 1). Most of the proteins identified were related to cell communication—a logical finding since they are contained in EVs and are transported to recipient cells.

Following this analysis, proteins FN1, ITGAV, VTN, ANXA2, and PFN1 were selected for validation based on their abundance in EV protein cargo and their important role in the implantation process. Immunoblotting of pHEEC EVs corroborated the presence of these proteins contained in EVs secreted by pHEECs (Fig. 5I).

## Discussion

The existence of EVs in a variety of reproductive tissues and biofluids, such as semen [13], follicular fluid [14], the oviduct [15], uterine fluid [16], and embryo-conditioned culture medium [17, 18], has raised the possibility that endometrial epithelial cells are also able to secret EVs that regulate molecular mechanisms involved in embryo implantation, suggesting a potential role of these EVs in reproduction. However, an optimized protocol to isolate EVs secreted by these cells is needed to study their function. To our knowledge, this is the first study to comprehensively evaluate three EV isolation methods on human endometrial epithelial cells in culture and to describe the proteomic content of EVs secreted by pHEECs from fertile women to define novel biomarkers of endometrial receptivity and implantation success.

While ultracentrifugation is the gold standard method for isolating EVs, it was never before compared with newer and less labor-intensive methods to isolate EVs from the extracellular medium of human endometrial epithelial cells. We compared the efficiency of ultracentrifugation against isolation using ExoQuick-TC reagent and the Norgen Cell Culture Media Exosome Purification kit. Ishikawa cells were used because they have characteristics of endometrial epithelial cells, they allow for in vitro culture of large cell numbers that secrete high amounts of EVs, and their controlled growth enables us to compare different experiments, contrary to primary cells that are more difficult to obtain and culture in vitro. While ultracentrifugation is based on a series of differential centrifugations that remove impurities and sediment EVs, the ExoQuick-TC reagent is based on the precipitation of EVs with hydrophilic polymers [30], and the Norgen kit is based on spin column chromatography that uses a silicon carbide resin separation matrix. Therefore, the population of EVs isolated with each methodology used will depend on the properties on which each method is based. To evaluate the efficiency of each method in isolating EVs, the size of EVs was analyzed by NTA. Although a similar size distribution of nanoparticles isolated was observed by the different methodologies (50–200 nm), a greater heterogeneity and smaller size was observed in nanoparticles isolated by the Norgen method, suggesting that the Norgen method is not able to isolate large nanoparticles. Cells produce and release different types of EVs, which can be divided into two different groups based on their biogenesis, exosomes (30–150 nm) and microvesicles (50–1,000 nm) [10]. However, there is no consensus in their size range, and the International Society for Extracellular Vesicles suggests subdividing EVs into small EVs (< 200 nm) and medium/large EVs (> 200 nm) [31]. Based on our findings, Ishikawa cells secrete mostly small EVs (< 200 nm) into the extracellular medium. The concentration of these nanoparticles was also analyzed by NTA and was lower in the Norgen group than in the other two groups, which is consistent with images obtained via TEM. In addition, analysis by TEM corroborated the presence of EVs with the typical cup-shaped morphology, caused by the contrast and dehydration of the samples before imaging, and showed a greater presence of artifacts in EVs isolated by ExoQuick-TC reagent.

Evaluation of the different protein markers characteristic of EVs in vesicles isolated from the Ishikawa culture media by the different isolation methods showed the presence of membrane organizers CD9 and CD81, which demonstrated the lipid bilayer structure of the EVs. Furthermore, cytosolic proteins HSP70 and TSG101 were analyzed to confirm the integrity of the EVs after isolation by the different methodologies, as the membrane encloses intracellular material [31]. Our results demonstrated that the expression of HSP70 and TSG101 was higher in EVs isolated by ultracentrifugation than in EVs isolated by the other two methodologies, in which expression was weak or even non-existent, suggesting that these methods do not maintain the integrity of isolated EVs. In this regard, our findings reinforce other studies in which HSP70 expression was weaker in samples obtained with precipitation solution-based methods like ExoQuick-TC than in samples obtained by centrifugation-based methods [32]. Expression of the other two protein markers, CD81 and CD9, was also higher in the ultracentrifugation group. The low expression of EV markers in the ExoQuick-TC and Norgen kit groups contrasts with protein quantification results, which showed that the ultracentrifugation group had a lower concentration of protein than the other two methodologies. This finding suggests a larger number of impurities co-isolating when using ExoQuick-TC reagent or the Norgen kit; this was also seen in our TEM analysis, in which the presence of artifacts was observed in EVs isolated by these methods. Based on these findings, ultracentrifugation remained the most efficient method for isolating EVs from conditioned culture medium of human endometrial epithelial cells because this method isolated a greater number of EVs with fewer artifacts, in line with use of this isolation method for different biofluids [32, 33]. Since ultracentrifugation isolates EVs based on their density/size, this technique efficiently isolated EVs regardless of the endometrial epithelial cell type, and was used to isolate EVs secreted by pHEECs.

EVs isolated by ultracentrifugation from pHEECs included both small and medium/large EVs, which was determined by the evaluation of EV protein markers and visualization by TEM. An optimal isolation method enabled us to describe the protein cargo of EVs secreted by pHEECs from women with proven fertility. Proteomic analysis revealed the presence of 218 proteins in these EVs that were enriched in biological processes related to embryo implantation, such as cell adhesion, differentiation, communication, migration, ECM organization, vasculature development, and reproductive processes. Eighty-two candidate proteins from EVs were selected based on their functional relevance in implantation success, classified into three groups based on their involvement in endometrial receptivity, embryo implantation processes, and early embryo development.

Several proteins identified from EVs were flagged as important for endometrial receptivity. Among these, the expression of annexins ANXA2, ANXA4, and ANXA5 are upregulated during the receptive phase of the menstrual cycle [34, 35]. ANXA2 is an adherent molecule between the embryo and the endometrial luminal epithelium during implantation and is involved in endometrial receptivity [36] and embryo development [37]. ANXA2 inhibition in endometrial stromal cells decreases embryo invasion in vitro, impairs decidualization, and contributes to the pathogenesis of severe preeclampsia [38, 39]. ANXA4 is implicated in apoptosis and is differentially expressed in endometrial cells during the window of implantation in women with a successful pregnancy compared to patients who do not achieve a pregnancy [40]. Collagen VI subunits α1, α2 and α3 are also present in this group, as they have been described to be localized in the endometrium of pregnant mouse uterus, and it has been observed a reduction of this protein after embryo implantation [41]. Some integrins, which are αβ adhesion receptors that mediate cell–cell adhesion and cell attachment to ECM proteins, are found in these EVs. Subunits α_1_, α_2_, and α_6_ usually associate with the β_1_ subunit, and this β_1_ subfamily of integrins is related to endometrial receptivity; they are present in the receptive phase of the menstrual cycle in stromal and epithelial cells and mediate cell adhesion to collagens and laminin, which are ECM components [42]. Mucins 1, 16, and 4 were also included in this group, and, although their role in embryo implantation is controversial for being anti-adhesive proteins, low levels of these proteins are associated with impaired receptivity of the endometrium [35, 43]. B2M, CD55, COMP, DPP4, F3, PROM1, and TGM2 were included in this group for being upregulated during the receptive phase of the menstrual cycle [35], and other proteins were included for having other roles related to endometrial receptivity, such as TLN1 for enhancing endometrial cell adhesion by regulating the Ras signaling pathway [44].

Among the proteins involved in the embryo implantation process itself, we found integrins ITGA3, ITGAV, and ITGB3. ITGA3 is expressed in embryos attached to endometrial cells in vitro and is not expressed in those that do not attach, along with other proteins included in this group, including CD44, THBS1, TNC, LAMA3, and ITGAV [45]. ITGAV is expressed during preimplantation development, and its candidate ligands include fibronectin (FN1) or vitronectin (VTN) [46]. Within this integrin family, integrin α_v_β_3_ is expressed in the endometrium and on the blastocyst at the time of implantation, and it is considered as a key component in the cascade of events leading to a successful implantation because it participates in attachment and embryo function [4, 47]. Laminin subunits LAMA3, LAMB3, and LAMC2 were also identified, and they form laminin 5, an ECM glycoprotein that binds to integrin α_3_β_1_ present in endometrial cells and preimplantation embryos, facilitating attachment [48]. FN1 is expressed on the surface of human hatched embryos, and it has been proposed to participate in embryo adhesion through its binding to endometrial CD26 [49] and in cell–cell communication during embryogenesis [50]. Furthermore, other studies showed that the α_5_β_1_ integrin is present on the apical surface of the blastocyst and adheres to ECM by binding to FN1 [51]. Profilin 1 (PFN1) is expressed in both blastocyst and endometrial epithelial cells and is a regulator of actin polymerization and the cytoskeletal network, which is necessary for an adequate embryo attachment in vitro [52]. VTN is another component of the ECM, which binds to integrin α_v_β_3_ previously mentioned, suggesting it also has a role in the embryo implantation process [53].

We also identified among EV cargos some proteins involved in early embryo development. Proteins in this group included ADAM10, CPM, GPX3, and UBA52. ADAM10 is a metalloproteinase that may moderate tight junctions in trophoblastic cells, which is an essential component for the development of the embryo to the blastocyst stage [54]. CPM is a cell surface peptidase expressed in trophoblastic cells, which is involved in its differentiation and function [55]. GPX3 is increased during embryo development and plays a role in processes involved in embryo development [56]. As such, GPX3 is proposed as a biomarker of embryo quality for polycystic ovary syndrome patients [57]. Finally, UBA52 is a ubiquitin ribosomal fusion protein that is involved in blastocyst formation, and its depletion causes developmental arrest at the 4-cell to 8-cell stage in porcine embryos [58].

## Conclusions

EVs secreted by pHEECs collected from fertile women and cultured in vitro can be efficiently isolated by ultracentrifugation, and their protein cargo implicates these EVs in biological processes related to endometrial receptivity, embryo implantation, and early embryo development. The identified proteins could define novel biomarkers of endometrial receptivity and implantation success.

## Data Availability

All data generated or analysed during this study are included in this published article.

## Abbreviations

DMEM: Dulbecco’s modified Eagle medium
EVs: Extracellular vesicles
FDR: False discovery rate
FBS: Fetal bovine serum
GO: Gene Ontology
LC–MS/MS: Liquid chromatography–tandem mass-spectrometry
NTA: Nanoparticle tracking analysis
pHEECs: Primary human endometrial epithelial cells
SD: Standard deviation
TEM: Transmission electron microscopy
WB: Western blotting
